# Identification in silico and expression analysis of a β-1-4-endoglucanase and β-galactosidase genes related to ripening in guava fruit

**DOI:** 10.1186/s43141-021-00289-x

**Published:** 2022-01-03

**Authors:** Mario A. Mejía-Mendoza, Cristina Garcidueñas-Piña, José S. Padilla-Ramírez, Ruth E. Soria-Guerra, José Francisco Morales-Domínguez

**Affiliations:** 1 Departamento de Química, Centro de Ciencias Básicas, Universidad Autónoma de Aguascalientes (UAA), Av. Universidad, #940, Ciudad Universitaria, C.P. 20100 Aguascalientes Aguascalientes, México; 2 Instituto Nacional de Investigaciones Forestales, Agrícolas y Pecuarias. Campo Experimental Pabellón, KM 32.5. Carretera Aguascalientes-Zacatecas, C.P. 20660 Pabellón de Arteaga, Aguascalientes, Ags México; 3 Facultad de Ciencias Químicas, Universidad Autónoma de San Luis Potosí (UASLP), Av. Dr. Manuel Nava No. 6–Zona Universitaria, C.P. 78210 San Luis Potosí, S.L.P. México

**Keywords:** *Psidium guajava*, Cell wall, Expression analysis, Bioinformatic analysis, Cell elongation, Fruit softening

## Abstract

**Background:**

Guava fruit softening is a crucial process during ripening and this process involves a number of enzymes that modifies the cell wall. Two of the enzymes that regulate this process are (a) the β-1, 4-endoglucanase 17 (BEG) which hydrolyze β-1, 4 bonds from cellulose and hemicellulose, and (b) β-galactosidase (BGA) that hydrolyzes pectin chains. Bioinformatics and expression analysis information on these genes is limited in guava fruit.

**Results:**

A fragment of a β-1, 4-endoglucanase 17 (*PgE17*), and another of a β-galactosidase (*PgGa1*) were identified. These sequences have a similarity of more than 85% with those reported in the NCBI database. In the guava genome, one homologous sequence was found for *PgE17* in Chr 4 and two homologous to *PgGa1*: one in Chr 3 and the other one in Chr 6. Putative protein PgE17 contains part of the glyco_hydro_9 domain. Putative protein PgGa1 has a part of the glyco_hydro_35 domain. Phylogenetic analysis of PgE17 and PgGa1 revealed that both are highly conserved inside the *Myrtaceae* family. In silico expression analysis showed that both PgE17 and PgGa1 work in a coordinated way with other cell wall modifier enzymes. Expression of these genes was found in all the guava samples analyzed. However, the highest expression was found in the fruit in the breaking and ripe states.

**Conclusions:**

A β-1, 4-endoglucanase 17, and β-galactosidase 1 sequences were identified. *PgE17* and *PgGa1* are expressed in all the plant tissues, and fruit ripening states. Although, the highest expression was on breaker and ripe states.

**Supplementary Information:**

The online version contains supplementary material available at 10.1186/s43141-021-00289-x.

## Background

The guava plant (*Psidium guajava* L.) belongs to the *Myrtaceae* family, and it grows on tropical and sub-tropical areas around the globe. Guava fruit has important nutritional properties like the high content of C vitamin of approximately 80–100 mg per gram of fruit [1]. Also, it is rich in tannins, tri-terpenes, flavonoids, saponins, lecithin, potassium, and soluble fiber [2]. In México, the guava fruit is one of the most important crops, and the most cultivated cv is the “media china”; which is characterized by its pale pink pulp color, intense sweet flavor, and significant commercial value due to its high demand on food and medicinal products [1]. Recent studies shows that ripening related enzymes and mechanical stresses during post-harvest period alters the cell wall (CW) structure damaging the fruit [3].

Due to the climacteric nature of guava fruit, its ripening process is regulated by their breathing levels and ethylene production [4]. Ethylene is the principal autocatalytic hormone in climacteric fruits, it is responsible for synthesizing ripening related enzymes [5]. Most of these enzymes work together in a coordinated way to modify and degrade complex carbohydrates to simple monosaccharides in the CW [6]. This process alters the firmness of the fruit (softening), as well as its flavor, and fosters the generation of volatile organic compounds (odor) [7].

Fruit softening is mainly achieved by the CW modification, principally in its primary wall pectin and hemicellulose residues [8]. The main enzyme that modify these residues is the β-1,4-endoglucanase (BEG) (EC 3.2.1.4) which hydrolyze β-1,4 bonds from cellulose and hemicellulose [9]. This enzyme acts during the breaker and ripe states of ripening when the pectin bonds are stronger, and many cellulose and hemicellulose residues are free. Another CW modifier enzyme is the β-galactosidase (BGA) (EC 3.2.1.23), which modifies pectin chain structure [10]. This enzyme can be found in three isoforms (I, II, and III), the isoform I is the most related to the ripening process [11]. Its function is to de-ramify pectin chains and the galactans and galactan-pyranoses degradation [10]. Its activity increases during the breaker and ripe ripening periods [12].

In this study, two gene fragments belonging to the *BEG* (*PgE17*) and *BGA* (*PgGa1*) enzymes in guava fruit were identified and characterized. In addition, the in silico and expression analysis of these two gene fragments in different parts of the guava plant (leaves, stem, and root), and in the green, breaker, ripe, and overripe ripening states of the fruit were performed.

## Methods

### Plant material

**Fig. 1:**
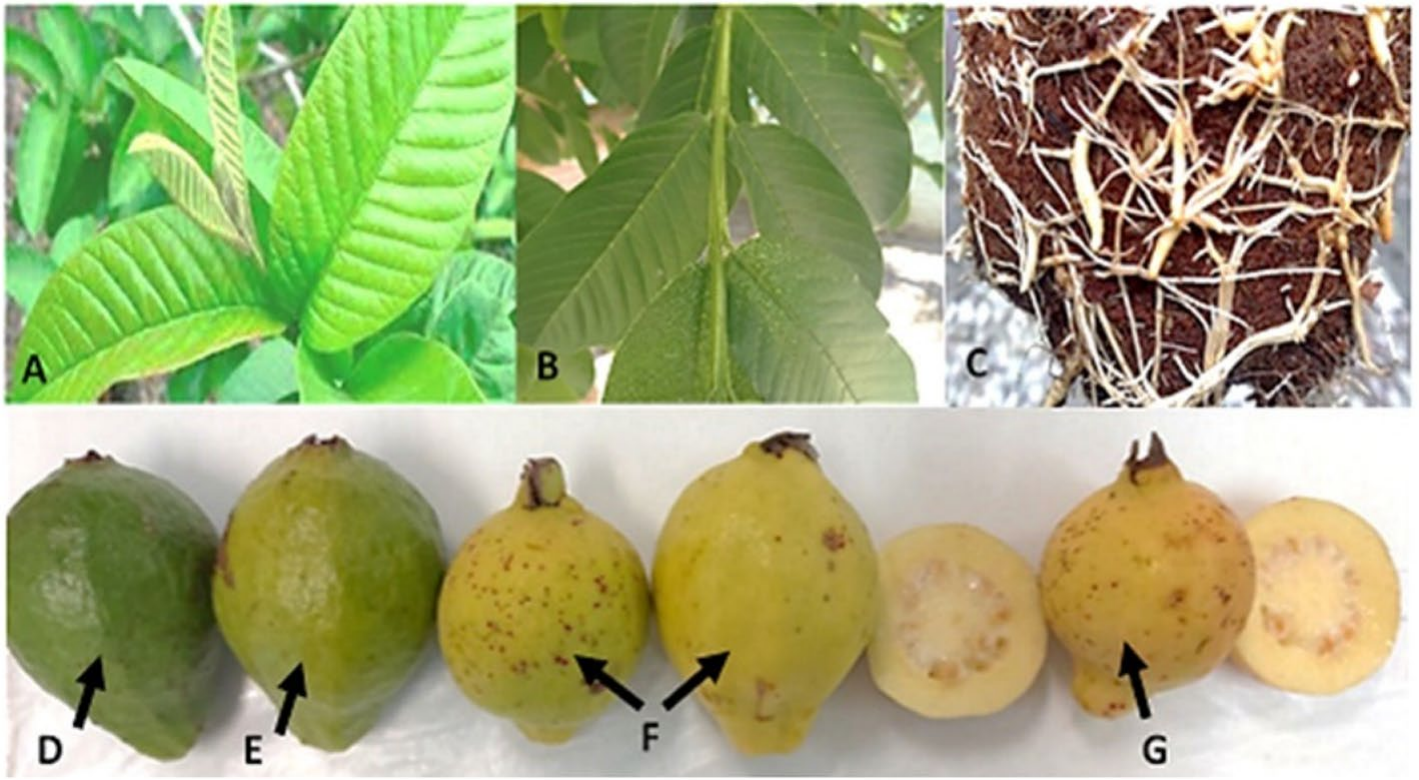
Sample tissues and ripening states of the guava fruit. A) young green leaves, B) young stem, C) root, D) green state guava fruit; 25 days after floriation, E) breaker state guava; three and a half months after floriation, F) ripe state guava fruits five months after floriation, G) overripe fruit six months after floriation

Samples of young leaves, stem, root, and four ripening states of the fruit: green, breaker, ripe, and over ripe (Fig. 1) were analyzed. They were taken from a guava tree c.v. Calvillo S-XX1 [2], located in the Germplasm Bank from the Experimental Site “Los Cañones”, belonging to the INIFAP, in the Huanusco municipality, state of Zacatecas, México (latitude 21°44′43.6 N; longitude 102°58′02.0 W).

### RNA extraction, RT-PCR, and cloning

Plant material was frozen and powdered with liquid nitrogen, RNA extraction was carried out following the protocol of Doyle and Doyle [13]. cDNA synthesis was performed with the SuperScript® III One-Step RT-PCR with Platinum® Taq kit (ThermoFisher®). Degenerated oligonucleotides were designed by aligning five different BGA and BEG aminoacidic sequences from taxonomic close plants respectively using the CODEHOP-J program from Viral Bioinformatics Research Center (https://4virology.net/virology-ca-tools/j-codehop/) [14]. Aminoacidic sequences from *Rhodamnia argentea* (XP_030541685.1 and XP_030543190.1), *Eucalyptus grandis* (XP_010049746.2 and XP_010069895.2), *Sysygium oleosum* (XP_030443911.1 and XP_030454903.1), *Quercus suber* (XP_023914075.1 and XP_023907247.1), and *Durio zibethinus* (XP_022732674.1 and XP_022770876.1), were used to design the oligonucleotides for *PgE17* and *PgGa1* (from *BGA* and *BEG*, respectively). Primers for the *PgE17* were forward: 5′-GACAACACCTTCGGCtgggayaa-3′, and reverse 5′-GGCCGGCTCGGACTGytcrtartc-3′; and for *PgGa1*: forward 5′-CAGACCTACGTGTTCtggaaygg-3′, and reverse 5′-GAAGTTGGTGCCGCCrtgrtacat-3′. The following PCR programs were used to amplify the fragments: 95 °C for 10 min, 35 cycles of 95 °C for 1 min, 56 °C, (for PgE17) or 52.4 °C (for PgGa1) for 40 s and 72 °C for 90 s, and 72 °C for 10 min. A Palm-Cycler® (Corbett Research) thermocycler was used for this process. PCR products were loaded into a 1.5% agarose gel, stained with ethidium bromide, and visualized under UV light. Wizard® SV Gel and PCR Clean-Up System (Promega®) and pGEM-T Easy Vector Systems kits were used to purify and cloning the desired PCR products.

### Bioinformatic analysis of the sequences

The obtained sequences were compared with the *Eucalyptus grandis* genome (Accession number: GCA_016545825.1) and the *P. guajava* genome assembly guava_V11.23 (Assembly accession number: GCA_016432845.1) [15] in the NCBI database using both nucleotide and aminoacid sequences by the BLASTn, BLASTp and TBLASTN webtools (NCBI, http://www.ncbi.nlm.nih.gov). Virtual translation of putative proteins was made using the Translate tool (https://web.expasy.org/translate/). The isoelectric point, the instability index; the aliphatic index and the GRAVY value of the putative proteins and their homologous regions found in BGA and BEG from *E.* grandis were measured with the ProtParam tool (https://web.expasy.org/protparam/) [16] from the ExPASy website. The CLUSTAL Ω (https://www.ebi.ac.uk/Tools/msa/clustalo/) [17] program was used for developing multiple alignments. Phylogeny analysis was performed with the PAUP4 software using: the distance method with DNA/RNA distances uncorrected p, unweighted least squares, negatives branch lengths in zero, all substitutions to the estimate count, output precision for scores up to 5 decimals, and a bootstrap of 100. Conserved domains and motifs search was performed using PgE17 and PgGa1 as search templates on the pFAM website (https://pfam.xfam.org/) [18]. BGA and BEG from *E. grandis* were used as a biological model for the co-expression analysis in the STRING platform (https://string-db.org/) [19].

### Expression analysis of *PgE17* and *PgGa1*

The expression of *PgE17 and PgGa1* was analyzed in leaf, stem, and root samples, as well as in fruit in the green, breaker, ripe, and overripe stages of ripening. The ribosomal 25S sub-unit gene from *Nicotiana tabacum* leaf was used as a reference gene to measure the relative expression as described in other works [20, 21]. The green ripening state results were used for data normalization.

The qPCR primers for *PgE17* were forward 5′-GGTACGGTCGTGCTGGTC-3′, reverse 5′-GGCGAAGATCCAGTGCTC-3′, and for *PgGa1*: forward 5′-AAAGGTTCACGCAGAAGATAGT-3′ and reverse 5’-CTGGTCCAAATTCGTTCTCTAT-3′. The qPCR reactions were performed using the Applied Biosystems™ Power SYBR™ Green PCR Master Mix kit, in a 96-well StepOne™ Real-Time PCR System (Applied Biosystems) thermocycler. Each reaction was assembled as follows: 10 μl of Power SYBR™ Green PCR Master Mix (2X), 200 ng of cDNA, and 100 nM of forward and reverse primers. The final volume was adjusted to 20 μl using Nuclease-Free Water. The PCR conditions were as follows, initial denaturation at 95 °C for 2 min, followed by 40 cycles of 5 s denaturation at 95 °C, an annealing and extension cycle at 52 °C for 30 s. Gathered data was analyzed using the StepOne™ Real-Time PCR System Software (Applied Biosystems). The relative fold changes in gene expression were calculated by the 2^−∆∆ct^ model.

## Results

### Bioinformatic analysis

A 331 nt sequence for *PgE17* and a putative translation protein sequence of 109 aa were obtained. The blastn and blastp analysis showed 91% similarity with several sequences reported in the NCBI data bases (Supplementary Figure 1). In *E. grandis* genome was found three *PgE17* homologous genes localized on chromosomes (Chr) 2, 4, and 8 (Supplementary Figure 2) with 89, 50, and 35% of similarity respectively (Supplementary Table 1). Likewise in the *P. guajava* genome there are one homologous sequence localized in the Chr 4 (9,959,984 to 9,960,316 nucleotide; Supplementary Figure 3) with a 98% identity (Supplementary Table 3).

**Fig. 2:**
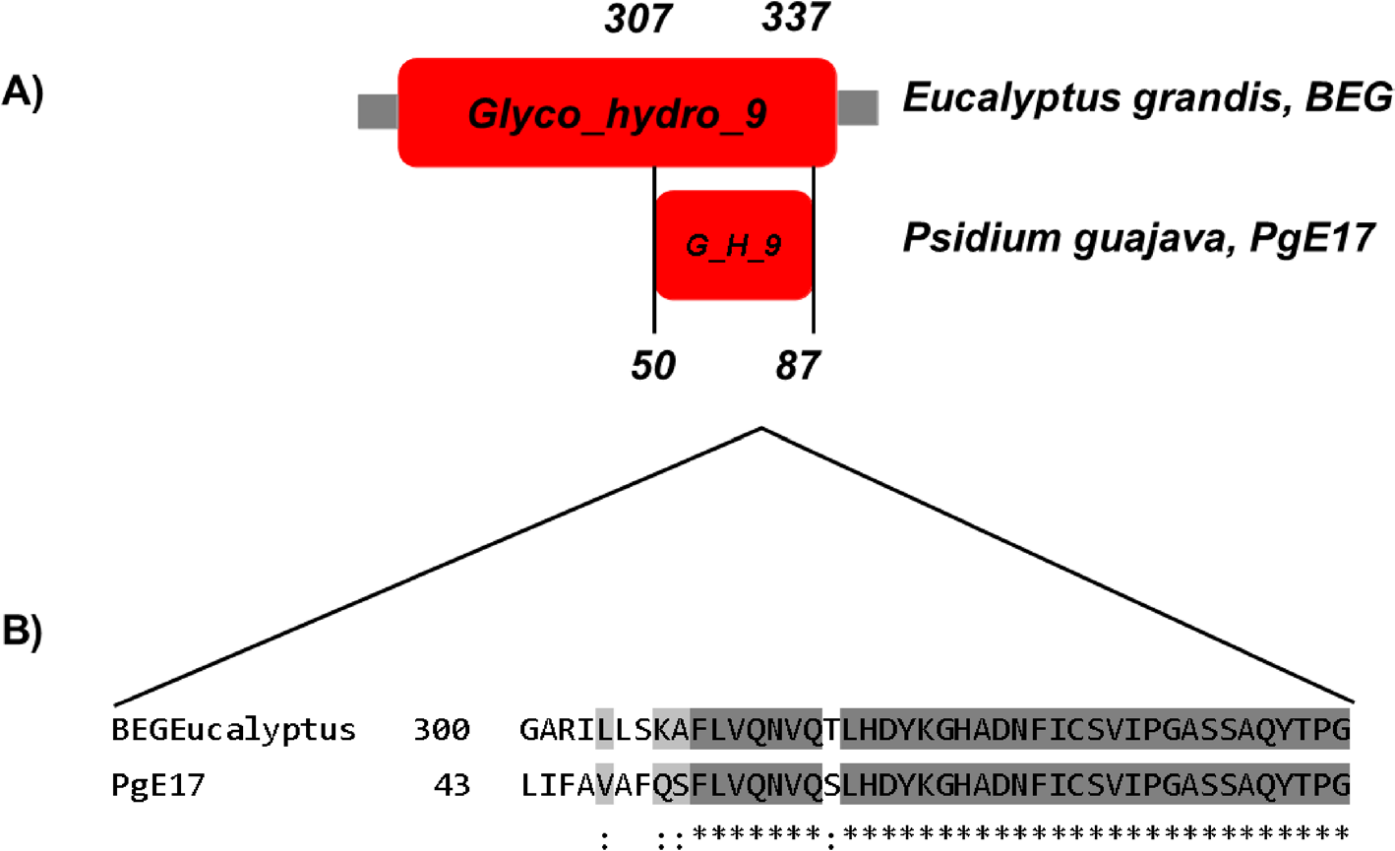
Graphic representation and alignment of the conserved domain between PgE17 and the BEG from *E. grandis*. **A** Glyco_hydro_9 conserved domain from the GH9 family, numbers indicate the position in each amino-acid sequence. **B** Alignment of the conserved domain. The blue square shows the characteristic PLN02266 domain of the GH9 family

**Fig. 3:**
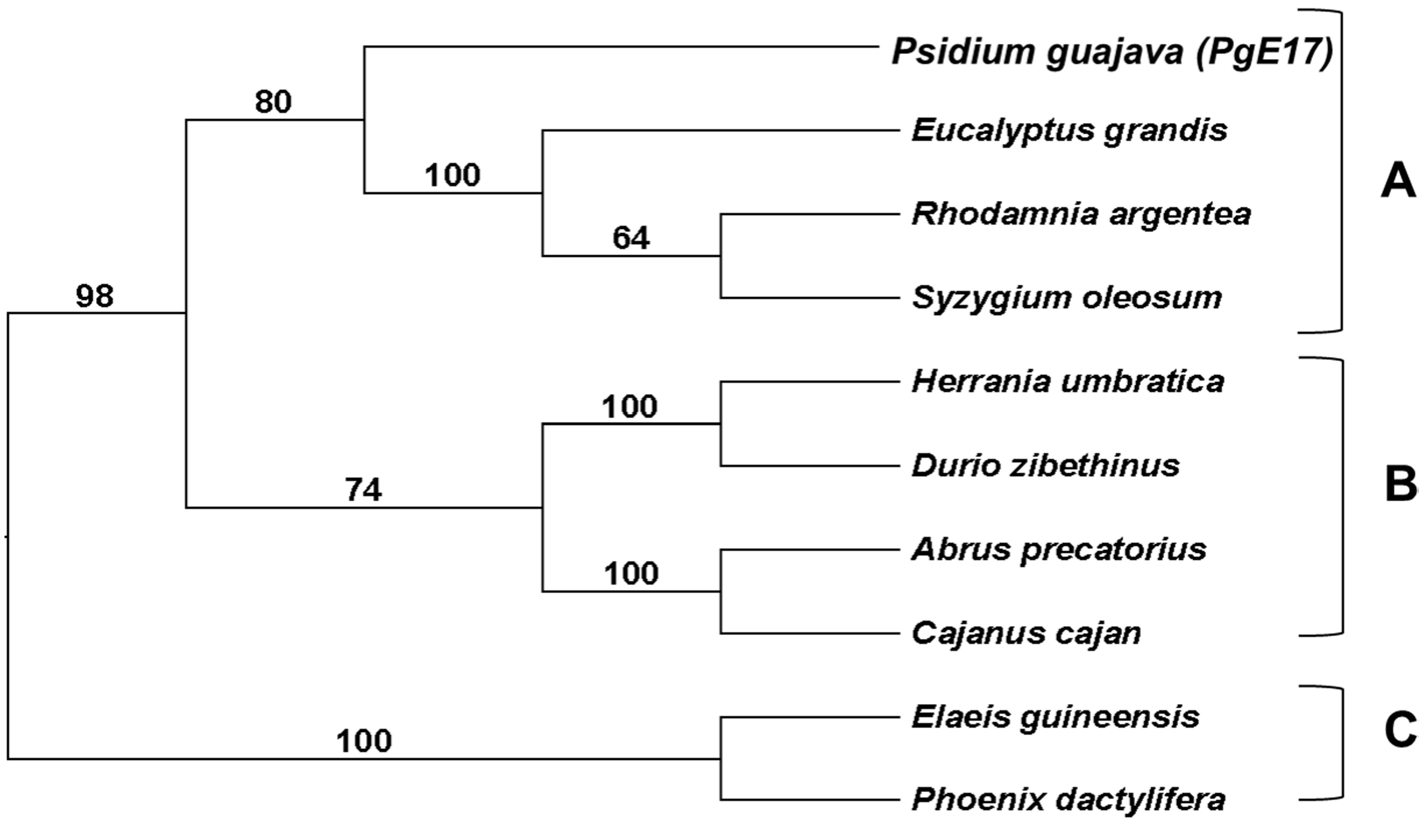
Distance dendrogram of PgE17 and nine BEG sequences of plants with a bootstrap of 100. Three nodes from different families are shown. A) *Myrtaceae* family where *PgE17* is included. B) *Rutaceae* and *Malvaceae* families*.* C) *Fabaceae and Arecaceae* families

PgE17 aa sequence possesses part of the conserved domain Glyco_hydro_9, also present in the BEG from *E. grandis* and *P. guajava* (Fig. 2). The phylogenetic analysis (Fig. 3) revealed that multiple significative differences exist between the 10 BEG aa sequences from different plants.

For *PgGa1*, a 750-nt-long sequence and 249 aa for its virtual translation were obtained. The blastn and blastp similitude analysis with BGA aa sequences from other plants showed an average similarity of 95 %. With *Rhodamnia argentea, Syzygium oleosum,* and *E. grandis* the similarity was 98 %, the highest obtained (Supplementary Figure 3). The similarity analysis of PgGa1 (Supplementary Figure 4) showed sequences with 71 and 92% of similitude in Chr 1, 3, 4, 6, 7, and 9 of *E. grandis* genome. However, a sequence found in the Chr 10 exhibited the highest similarity with PgGa1 with 98.2% (Supplementary Table 2).

Furthermore, PgGa1 blastn analysis on the *P. guajava* genome founded one identical sequence and one similar sequence on the Chr 6 and 3 respectively (Supplementary Figure 4). Identical sequence found in the Chr 6 is located between the nucleotides 38,077,142 to 38,077,288 and has a 100% identity with *PgGa1* (Supplementary Table 4). On the other hand, Chr 3 sequence was from nucleotide 7,575,108 to 7,575,192 and has 83.53% identity with *PgGa1* (Supplementary Table 4).

**Fig. 4:**
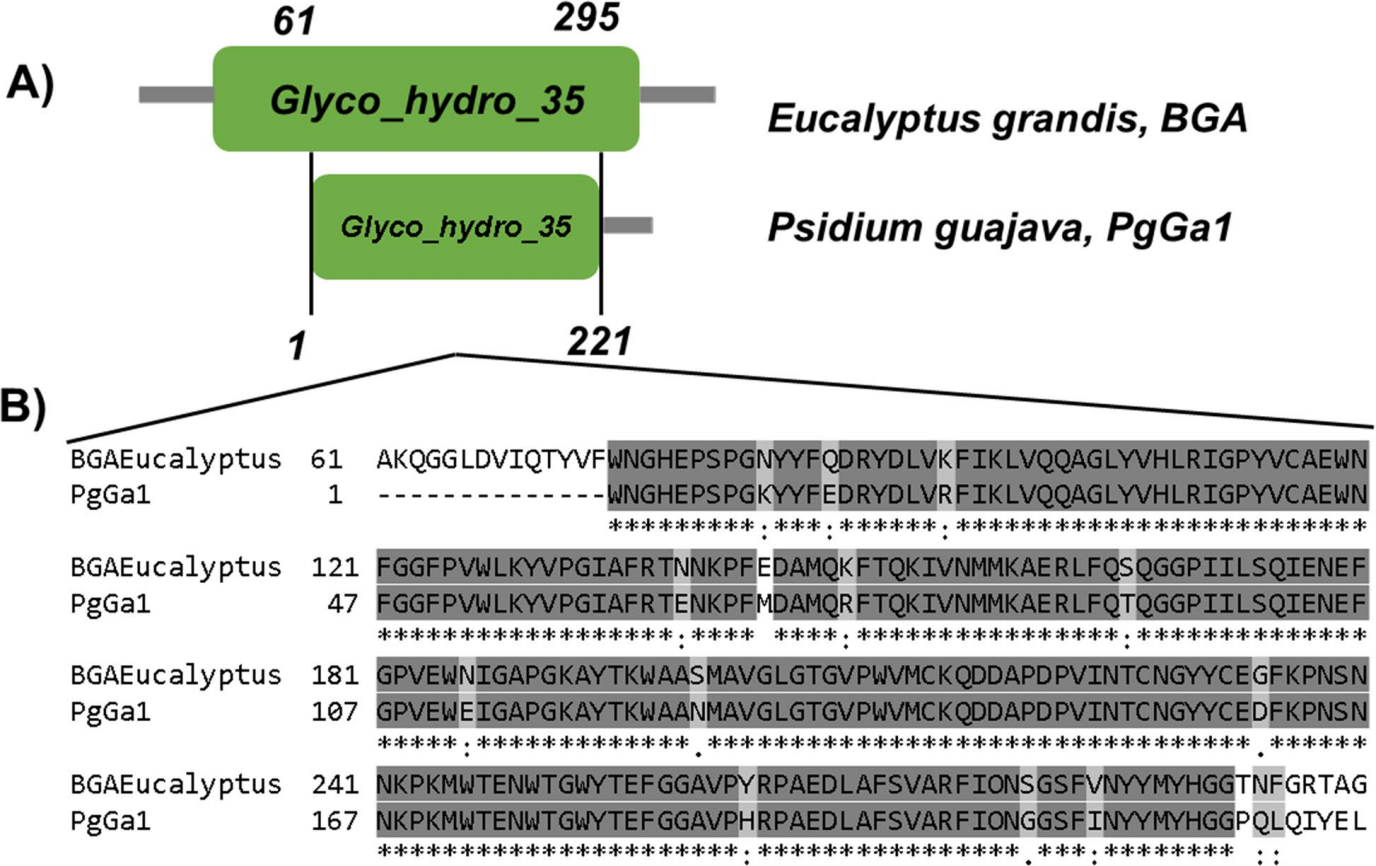
Graphic representation and alignment of the conserved domain between PgGa1 and the BGA from *E. grandis*. **A** Glyco_hydro_35 domain of the GH35 family in the *E. grandis* BGA and PgGa1, numbers indicate the amino-acid position in each sequence. **B** Alignment of the conserved region between the two enzymes, the black square shows the active site GGPIILSQIENEF

**Fig. 5:**
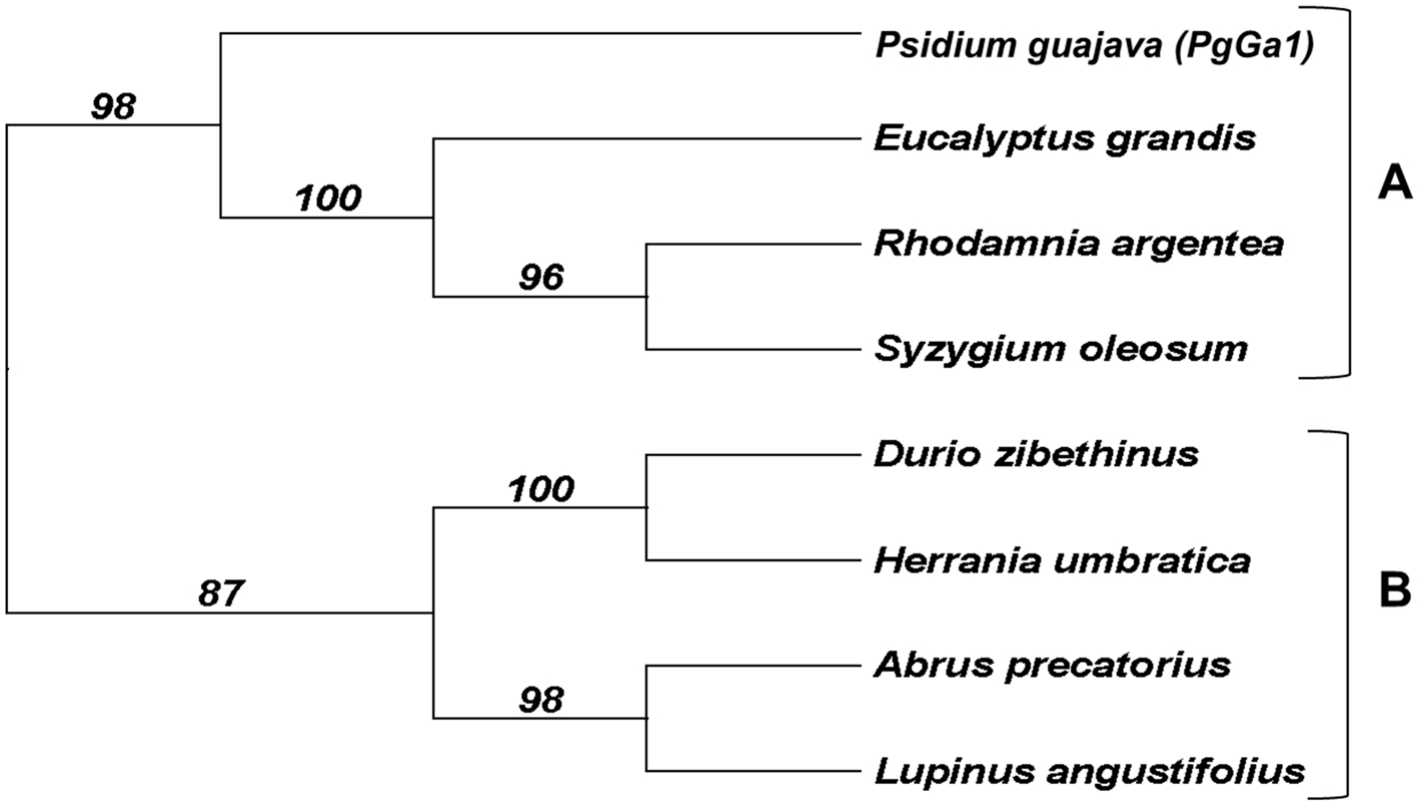
Distance dendrogram of PgGa1 and ten amino acid sequences of BGA with a bootstrap of 100. Two nodes are shown A) *Myrtaceae* members only, B) Contains both *Malvaceae* and *Fabaceae* members

In the putative aa sequence of PgGa1, the Glyco_Hydro_35 conserved domain was found, which is exclusive to the BGA and enzymes belonging to the family 35 of glycosyl hydrolases (GH35) [19]. This conserved domain is located between the 29 and 335 aa residue positions in the BGA of *E. grandis* (Fig. 4). PgGa1 also contains the putative active site GGPIILSQIENEF (Fig. 4, black square). The phylogenetic analysis revealed a minimal node formation and situated PgGa1 inside the *Myrtaceae* family (Fig. 5).

The parametric values showed in Table 1 were used to compare the physicochemical properties of the putative proteins of PgE17 and PgGa1 of *P. guajava* with the respective *E. grandis* proteins found in the database, both the complete protein and the corresponding conserved region.

**Table 1:** Parametric values of putative proteins PgE17, PgGa1 from *P. guajava*, BEG, and BGA from *E. grandis* and it conserved regions

Protein	Theoretical p.I	GRAVY	Aliphatic index	Instability index	Length (aa)
β-1,4-endoglucanase 17 conserved region from *E. grandis*	7.12	− 0.020	86.85	17.40	108
Conserved region in Chr 4 of *P. guajava* genome	6.28	0.274	94.55	37.89	101
PgE17	6.25	0.344	96.57	40.15	108
β-galactosidase 1 conserved region from *E. grandis*	5.95	− 0.336	62.79	33.68	247
Conserved region in Chr 6 of *P. guajava* genome	6.18	− 0.298	65.49	31.20	204
PgGa1	5.54	− 0.476	63.16	38.72	248

pI isoelectric point, GRAVY grand average of hydropathicity

### In silico co-expression analysis of BGA and BEG

**Fig. 6:**
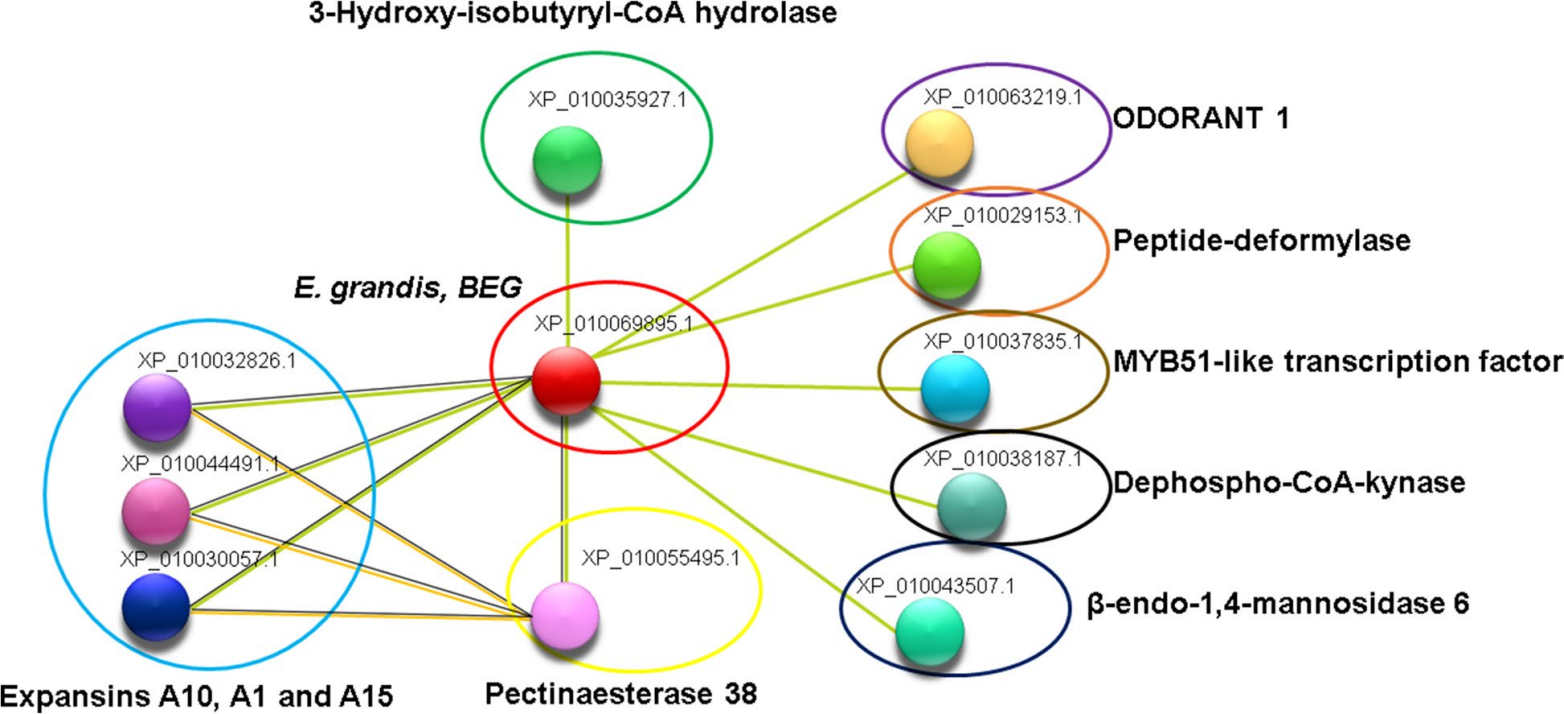
Co-expression analysis of the *E. grandis* BEG homologous to *PgE17*. Blue circle, expansins A10 (XP_010032826.1); A1(XP_010044491.1), and A15 (XP_010030057.1). Green circle, hydroxy isobutyryl-CoA hydrolase 3 (XP_010035927.1). Yellow circle, pectinesterase 38 (XP_010055495.1). Purple circle, ODORANT 1 (XP_010063219.1), orange circle, peptide deformylase (XP_010029153.1). Golden circle, MYB51 like transcription factor (XP_010037835.1), black circle, de phospho-CoA-kinase (XP_010038187.1). Blue navy circle, β-endo-1,4-manosidase 6 (XP_010043507.1). Yellow lines represent text mining relation; purple lines, experimentally determined relationship, and black lines show a direct co-expression between proteins

**Fig. 7:**
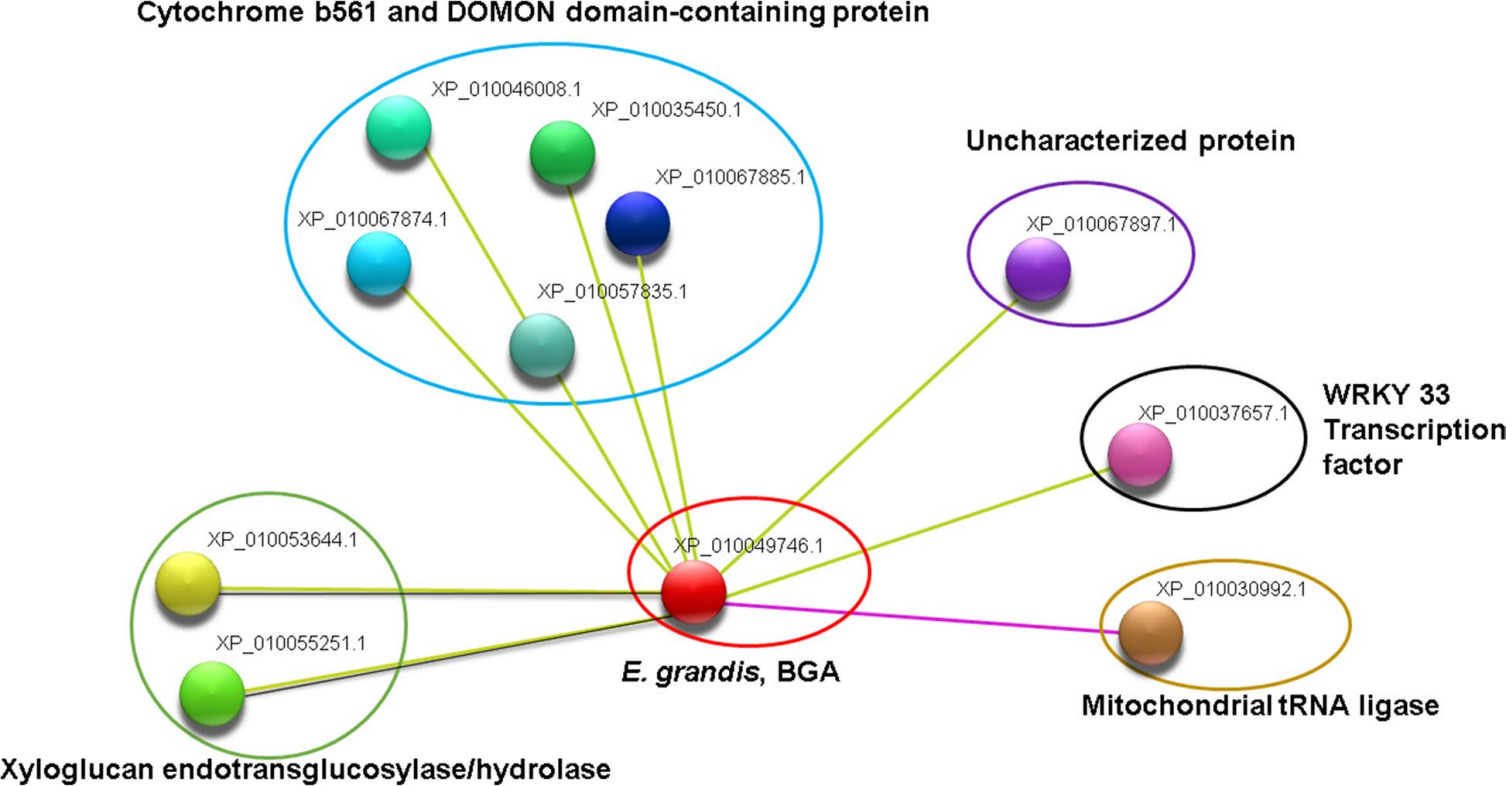
Co-expression analysis of the *E. grandis* BGA homologous to *PgGa1*. Blue circle, DOMON domain b561 proteins (XP_010046008.1), b561.2 (XP_010035450.1), b561.3 (XP_010067885.1), b561.4 (XP_010067874.1), and b561.5 (XP_57835.1). Purple circle, uncharacterized protein (XP_010067897.1). Black circle, WRKY 33 transcription factor (XP_010037657.1). Golden circle, tRNA mitochondrial ligase (XP_010030992.1). Green circle, XET/H1 (XP_010053644.1) and XET/H2 (XP_010055251.1). Yellow lines represent text mining relation, purple lines, experimentally determined relationship, and black lines show a direct co-expression between proteins

In silico co-expression analysis of *PgE17* displayed a direct relationship with CW modifiers proteins and proteins related to the synthesis metabolic pathways of aromatic compounds during the ripening process. CW modifiers proteins were three expansins, a β-endo-1,4-manosidase, and a pectinesterase. Besides, the aromatic compounds synthesis related were: a peptide deformylase, a de-phospho-CoA-kinase, a hydroxy-isobutyryl-CoA hydrolase, and a MYB51-like transcription factor (Fig. 6). In silico co-expression analysis for *PgGa1* (Fig. 7) showed the association with CW elongation enzymes during ripening such as two xyloglucan endotransglucosylase/hydrolase (XET/H) and to others that are indirectly related to the ripening process such as the DOMON-domain proteins from the b561 cytochrome, WRKY transcription factor, and a tRNA ligase.

### Expression analysis

**Fig. 8:**
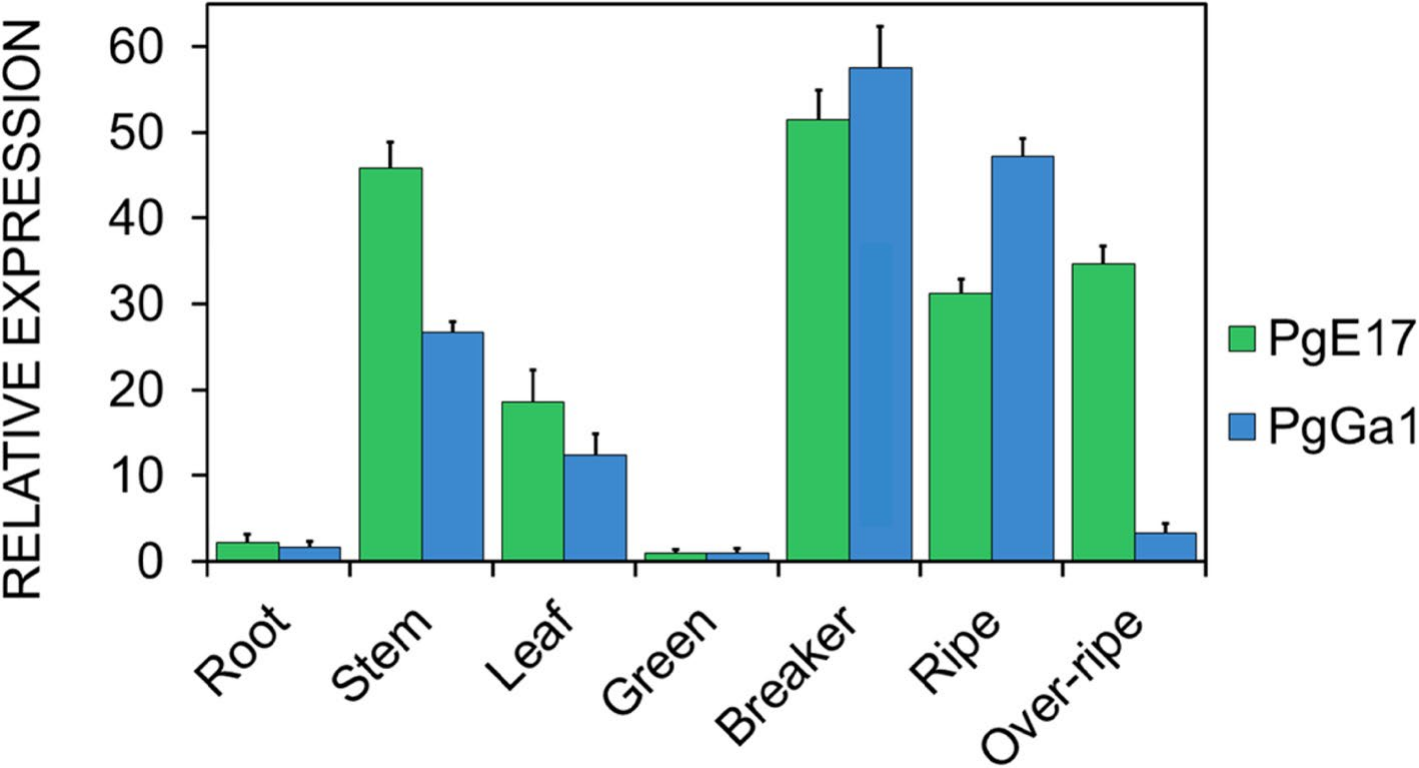
Expression analysis of *PgE17* (green) and *PgGa1* (blue). Expression levels were measured in root, leaves, and stem tissues in four ripening states. The green state was used as a reference for the relative expression

qPCR analysis for *PgE17* exhibited a higher expression on the breaker state in fruit, equivalent to 50 times more than in the green state. *PgE17* expression was between 15 and 20 times lower in ripe and over ripe fruits than breaker fruit (Fig. 8), the expression in the root was the lowest, with about 45 times lower than the breaker. Meanwhile, the expression of *PgE17* in stem was 40–45 times higher than the green fruit, being the second higher value. The expression of *BEG* in leaf was between 18 and 20 more times than in green fruit, the second lowest after root.

For *PgGa1* (Fig. 8), the highest expression was found in the breaker and ripe states, with 55 and 45 times more than in the green state, respectively. On the other hand, *PgGa1* expression in the over ripe state was 50 times lower than the ripe fruit. This is the lowest expression of all fruit states, and second lowest of all tissues, only two to three times higher than the expression observed in root. Expression levels in leaves and stem were between 20 to 30 times lower than in fruit, but higher than root; tissue in which expression was null.

## Discussion

Fruit ripening is mainly due to two different metabolic events: starch degradation and cell wall modification [22]. Referring to cell wall compounds, pectin polymers are responsible for the firmness in green fruits and their degradation during the ripening process leads to the softening of the fruit [12]. Like many fruits, guava fruit suffers from these process, several studies have been carried out on the enzymatic activity of both carbohydrate metabolism and the cell wall. The ability to modify the cell wall depends on the type of fruit and in many cases is not related to the softening rate, nor does it depend on the enzymes themselves [22].

There are several works in the literature about the enzymatic activity related to the ripening of the guava fruit, but there are few related to the expression of genes of *BEG* and *BGA*. Therefore, this work focuses on the identification and bioinformatic analysis and expression study in different organs of the guava plant and its comparison with the enzymatic activity reported in the literature.

The aa sequence of PgE17 contained a conserved region of the Glyco_hydro_9 domain belongs to the family 9 of the glycosylhydrolases (GH9) that modifies the CW indirectly [23], and also contains a high number of contiguous hydrophobic aa that helps shape its tridimensional structure, which is a general characteristic between the BEG enzymes [24].

Physicochemical properties show that PgE17 has different characteristics than the BEG of *E. grandis* and similar characteristics of the BEG of *P. guajava* [15]. The pI value, which is the pH at which the proteins have no electrical charge, was acidic for PgE17, while it was basic for *E. grandis* BEG (Table 1). The GRAVY value allows proteins to be classified as hydrophobic, if the value is positive, or as hydrophilic, if the value is negative. Based on the GRAVY values obtained (Table 1), the PgE17 sequence can be considered hydrophobic, like the transmembrane proteins. In contrast, both *E. grandis* BEG sequences can be considered hydrophilic proteins. The instability index measures the stability of the protein in water, and proteins are considered stable if this index is less than 40. Therefore, the instability index reveal that PgE17 is unstable, while the conserved region of BEG from *E. grandis* and *P. guajava* genome are stable (Table 1). These differences can be attributed to the fact that the aa sequence of the proteins of this family differs from one species to another [23]. The only value consistent across the different sequences was the aliphatic index. This parameter measures the volume occupied by the aliphatic side chains and it is indicative of thermostability, proteins with a high value are stable in a wide temperature range [16]. PgE17, the conserved region of BEG, and the complete BEG protein have large aliphatic index values, revealing their thermostability (Table 1). The aliphatic index of PgE17 was higher than that of the conserved region of BEG and that of the full BEG protein from *E. grandis* (Table 1)*,* indicating that PgE17 is more thermostable.

Phylogenetic differences between 10 BEG from plants including PgE17 were previously described by Jara and Castro [24], which they establish multiple differences between the amino acid sequences in BEG genes inside the GH9 family, which is derived from divergent evolution.

The PgGa1 sequence has similitude with a one sequence of the Chr 10 of *E. grandis* corresponding to BGA and with BGA *P. guajava* [15]. BGA has been found in *Rhodamnia argentea*, *Syzygium oleosum*, and *E. grandis,* all of them members of the *Myrtaceae* family. Glyco_hydro_35 domain found in PgGa1 changes the typical configuration of BGA to β-anomeric [25], allowing the hydrolyzation of the bond between galactose and glucose [26]. On the BGA enzymes active site GGPIILSQIENEF, is located right after the signal peptide, inside the N-terminal region [25] and acts in the p-nitrophenyl-β-D-galactopyranosides and 1,4-β-D-galactans residues from the CW modifications [27, 28].

Parametric analysis showed all sequences analyzed have similar values (Table 1). For example, the negative GRAVY values suggest that are hydrophilic, with a higher probability of being globular. These results, together with the similarity found in the phylogenetic analysis, support the idea that the proteins of this family are conserved in the different species [25].

Low diversification of nodes in the phylogenetic tree indicates that aa sequences of BGA are highly conserved between and inside plant species. A similar phylogeny with a low number of nodes of this protein was described by Smith and Gross [29] and explained by Tanthanuch et al. [25], who mentions that the BGA gene in plants has suffered a minimal number of evolutionary changes since the divergence of plants and animals.

*PgE17* co-expressed with several CW modifiers proteins, relates to ripening in the following ways: the expansins hydrolyze the hydrogen bonds from cellulose and hemicellulose, causing the CW expansion [30]; also there is evidence of their high expression on guava fruits [31]. The β-endo-1,4-mannosidase hydrolyze the β-1,4 intern bonds of mannose chains from the CW producing the characteristically softening during ripening [32]. Pectinesterase 38 de ramify homogalacturonan chains from the CW releasing hemicellulose and cellulose residues, boosting the softening of the fruit [3]. Peptide deformylase hydrolyses the N-formyl groups from immature protein sequences from chloroplast in plants [33]; malfunction of this enzyme causes the accumulation of misfolded proteins and chlorosis, resulting in bad development and malformation of the plant tissues, including the fruit [34]. The de-phospho-CoA-kinase removes phosphate groups from the phosphorylated CoA groups for the biosynthesis of the A-Coenzyme, which is essential for plant gas exchange during ripening of the fruit [35]. The MYB51-like transcription factor related to the ripening process is significant because it acts as a callose transporter to the CW [36]. Increasing callose concentrations in the CW and phloem have been observed during the ripening of grapefruit (*Vitis vinifera)* [37]. Regarding aromatic compounds, it has been observed that hydroxy-isobutyryl-CoA hydrolase takes part in their synthesis during the ripening of peach fruit (*Prunus persica* cv. Hujiingmilu) [38]; as well as for the biosynthesis of fat acids [39], which have been related to the ripening of the olive fruit (*Olea europea* cv. Picual) [40, 41]. The ODORANT1 factor, together with the EOBII transcription factor regulates the production of aromatic compounds in flowers and during the ripening of some fruits like tomato (*Solanum lycopersicum*), strawberry (*Fragaria vesca*), and the blueberry (*Vaccinium corymbosum*) [42].

*PgGa1* co-expresses with ripening involved proteins such as, XET/H enzymes that induce hydrolysis or addition of xyloglucan chains to the CW, giving extensibility to the cell, which is a necessary process for ripening [43]. Furthermore, PgGa1 is indirectly related to ripening proteins, like those with the DOMON-domain b561, which catalyzes the electrons trans-membranal transport, and the ascorbate regeneration [44]. This could be related to the high amount of C vitamin in the guava fruit [1]. WRKY and RAP2 transcription factors has been observed to act altogether in cellular signaling processes and abiotic and biotic stress responses in guava fruit [45, 46]. High tRNA-met expression levels has been found during the ripening of the palm fruit (*Phoenix dactylifera*) and tomato [47]. Methionine is the main precursor of ethylene, an essential compound in climacteric fruit ripening [48].

The expression analysis of *PgE17* showed a high expression in the breaker period and is could be due because in this state there is an increasing rate of growing and development of the fruit, and the rupture of large amounts of β-1,4 bonds from hemicellulose is necessary, in which BEG enzymes are involved [49]. Similar results were described by Carey et al., who found high BEG expression levels in tomato fruit in the breaker state [28]. Besides, the decrease in the subsequent states (ripe and over ripe) is caused by the previous accumulation of BEG which caused a depletion of its substrate [50]. This was confirmed in tomato fruit and strawberry by measuring the expression and quantity of BEG enzymes during the breaker, ripe and overripe states, resulting in high concentrations of BEG enzyme during the breaker state and a decrease in its activity in the ripe period [51, 52]. And the other hand, the low expression of *PgE17* in root concords with Buchanan et al. [9], who demonstrated in sorghum (*Sorghum bicolor*), maize (*Zea mays*), rice (*Oryza sativa*), and some pasture (*Brachypodium* sp.), that BEG expression levels depend on the specie and type of root, among which the root does not need significant modifications on its CW. *PgE17* expression in stem and leaves is high due to their constant growth and development rate, BEG enzymes participate in the rearrangement of these structures [50].

In *PgGa1* the high expression on breaker and ripe state maybe due to the onset of fruit softening in this period, and so it requires the de ramification of pectin chains that gives support and stiffness to the CW [3, 10]. These events have been seen in the enzymatic activity of BGA in these states generates a constant amount of hemicellulose residues [6, 53], which is related to the high expression of *PgE17* found in braker state. This theory is confirmed by Zainon et al. [11], who analyzed the BGA and BEG activity and substrate in mango fruit (*Mangifera indica* cv. Harumanis)*,* proved that in the breaker and ripe states the decrease in pectin chains caused by BGA enzymes resulted in a reduction of hemicellulose chains due to BEG activity. Significant decrease of *PgGa1* expression in the over ripe state is because it is limited to the pectin available chains [10]. The last ripening state exhibits a low quantity of these chains due the action of other enzymes, such as pectinesterases [3] and polygalacturonases [53] that act in response to the massive release of pectin residues caused by BGA. Together, these results indicate that *BGA* high expression plays a key role in the CW degradation, process which have been stablished as an essential contributor of the softening of the fruit during ripening [15].

The low expression of *BGA* observed in stem, leaves and roots, suggests that the CW of these tissues does not need significant modifications. Similar results were reported in apple (*Malus domestica*) [54].

## Conclusion

Two gene partial sequences were identified in the guava plant: *PgE17* and *PgGa1*. Bioinformatic analysis showed that *PgE17* belongs to a *BEG*, and *PgGa1* to a *BGA* gene, and that they have homologous in the *Myrtaceae* family. The *q*PCR analysis revealed that the expression of both genes increases in the breaker and ripe stages, which demonstrates the importance of these enzymes in the softening and ripening processes of the fruit.

## Supplementary Information

**Additional file 1.**

## Data Availability

The datasets generated during and/or analyzed during the current study are not available, only the sequences in the GenBank accessions, but are available from the corresponding author on reasonable request.

## Abbreviations

CW: Cell wall
BEG: β-1,4-endoglucanase
BGA: β-galactosidase
LANBAMA: Laboratorio Nacional de Biotecnología Agrícola y Ambiental
IPICyT: Instituto Potosino de Investigación Científica y Tecnológica
GH9: Family 9 of the glycosylhydrolases
*E. grandis*: *Eucalyptus grandis*
GH35: Family 35 of glycosyl hydrolases
XET/H: Xyloglucan endotransglucosylase/hydrolase
tRNAmet: tRNA methionine
