# Supplementary material for: Identification in silico and expression analysis of a β-1-4-endoglucanase and β-galactosidase genes related to ripening in guava fruit
Source: J Genet Eng Biotechnol. 2022 Jan 3;20:3. doi: 10.1186/s43141-021-00289-x (PMC8724366; doi:10.1186/s43141-021-00289-x)
Supplement: Supplementary file 1 — Additional file 1. [file 43141_2021_289_MOESM1_ESM.docx]

**Supplementary table 1**. Similar sequences to PgE17 found in *E. grandis* genome

| **Chromosome** | | **Position (nt)** | **Gene** | **Similarity % with *PgE17*** |
| --- | --- | --- | --- | --- |
| 8 | 12681226-12684786 | | β-1,4-endoglucanase 1 | 89.0 % |
| 4 | 30218926-30221748 | | β-1,4-endoglucanase | 51.6 % |
| 2 | 48242919-48248035 | | β-1,4-endoglucanase 4 | 35.4 % |

**Supplementary table 2**. Similar sequences to PgGa1 and PgE17 found in *P. guajava* genome

| **Chromosome** | | **Position (nt)** | **Gene** | **Similarity % with *PgE17* or *PgGa1*** |
| --- | --- | --- | --- | --- |
| 6 | 38,076,354 - 38,077,943 | | β-galactosidase 1 | 100% |
| 3 | 7,575,108 - 7,575,192 | | β-galactosidase 1 | 83.63% |
| 4 | 9,959,984 - 9,960,316 | | β-1,4-endoglucanase 17 | 98.20% |

**Supplementary table 3**. Similar sequences to PgGa1 found in *E. grandis* genome

| **Chromosome** | | **Position (nt)** | **Gene** | **Similarity % with *PgE17*** |
| --- | --- | --- | --- | --- |
| 10 | 34195225-34202265 | | β-galactosidase 1 | 98.2 % |
| 9 | 33617356-33621921 | | β-galactosidase 11 | 88.2 % |
| 7 | 32601186-32606779 | | β-galactosidase 5 | 92.4 % |
| 6 | 43199799-4320781 | | β-galactosidase 1 | 85.9 % |
| 4 | 12963708-12970304 | | 1,3-β-galactosidase | 72.8 % |
| 3 | 74986360-74991922 | | β-galactosidase 1 | 85.1 % |
| 1 | 27539469-27543506 | | β-galactosidase 15 | 71.2 % |


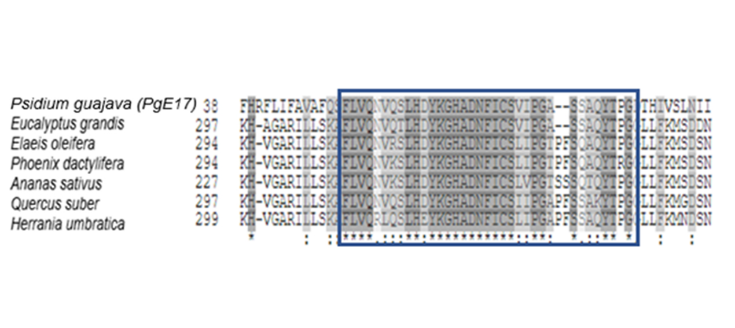


**Supplementary Fig. 1** Multiple aa alignment of PgE17 against other 6 sequences of BEG from plants. The blue square shows the conserved motif inside the PLN02266 domain, characteristic of the GH9 family.


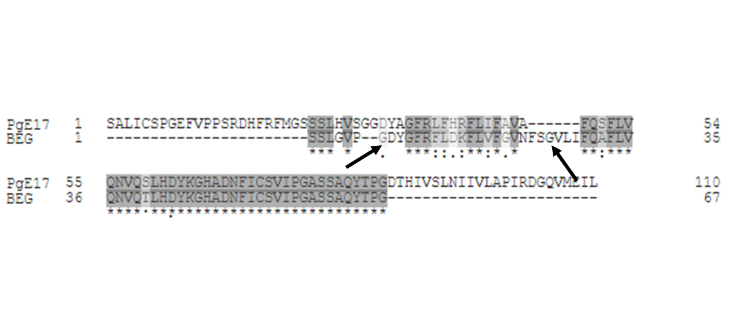


**Supplementary Fig. 2** Alignment of aminoacidic PgE17 sequence against *E. grandis* Chr 8 in the +2/-2 ORF. Black arrows show gaps inside both sequences.


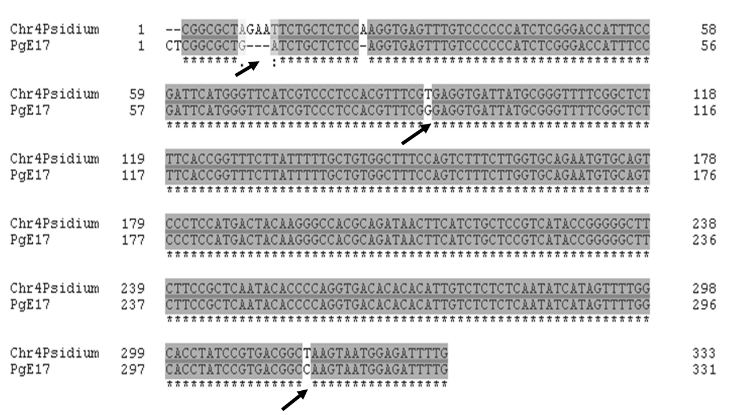


**Supplementary Fig. 3** Alignment of nucleotide PgE17 sequence against *P. guajava* Chr 4 assembly. Black arrows show gaps inside the alignment


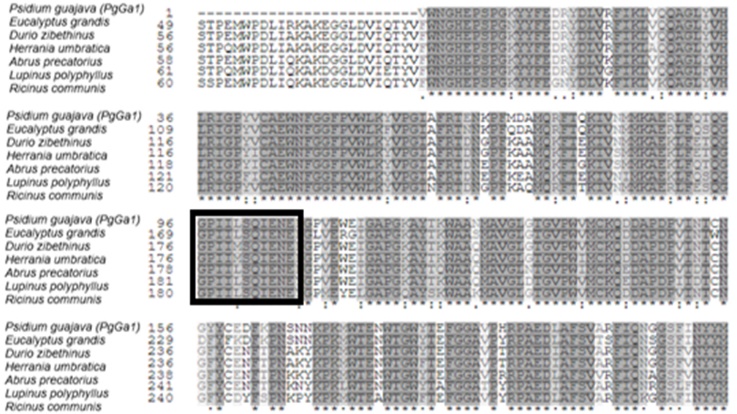


**Supplementary Fig. 4** Multiple aminoacidic alignment of PgGa1 and 6 BGA sequences from plants. The black square shows the active site “GGPIILSQIENEF”.


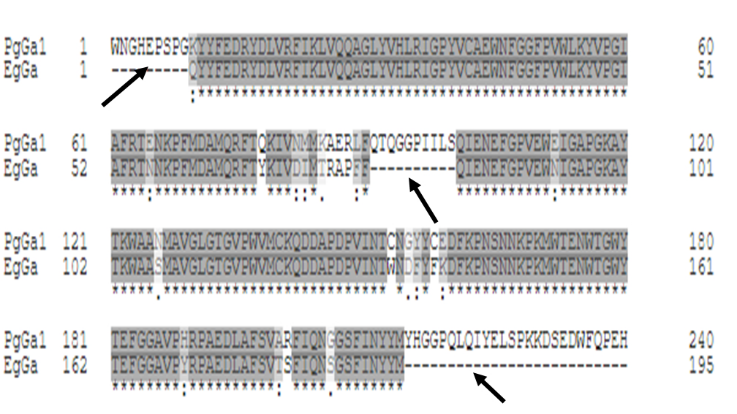


**Supplementary Fig. 5** Alignment of aminoacidic PgGa1 sequence and *E. grandis* Chr 10 in the +1/+3 ORF. Black arrows show gaps inside both sequences.


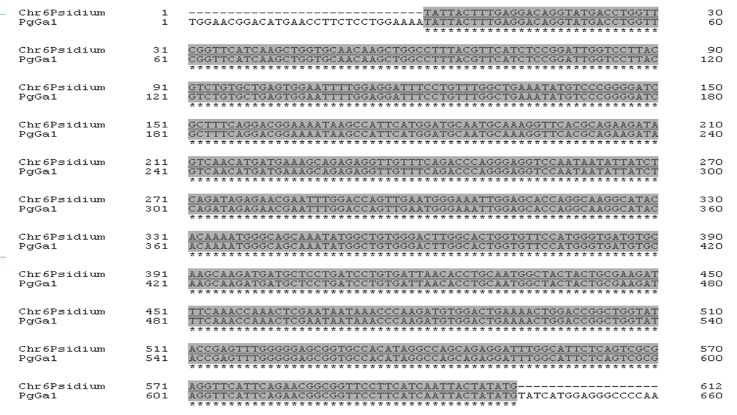


**Supplementary Fig. 6** Alignment of nucleotide PgGa1 sequence against *P. guajava* Chr 6 assembly.
